# Admixture mapping reveals loci for carcass mass in red deer x sika hybrids in Kintyre, Scotland

**DOI:** 10.1093/g3journal/jkab274

**Published:** 2021-08-02

**Authors:** S Eryn McFarlane, Josephine M Pemberton

**Affiliations:** 1 Institute of Evolutionary Biology, School of Biological Sciences, University of Edinburgh, Edinburgh EH9 3FL, UK; 2 Department of Biology, Lund University, 22100 Lund, Sweden

**Keywords:** admixture mapping, hybridization, carcass mass, *Cervus elaphus*, *C. nippon*

## Abstract

We deployed admixture mapping on a sample of 386 deer from a hybrid swarm between native red deer (*Cervus elaphus*) and introduced Japanese sika (*Cervus nippon*) sampled in Kintyre, Scotland to search for quantitative trait loci (QTLs) underpinning phenotypic differences between the species. These two species are highly diverged genetically [*F*_st_ between pure species, based on 50K single nucleotide polymorphism (SNPs) = 0.532] and phenotypically: pure red have on average twice the carcass mass of pure sika in our sample (38.7 kg *vs* 19.1 kg). After controlling for sex, age, and population genetic structure, we found 10 autosomal genomic locations with QTL for carcass mass. Effect sizes ranged from 0.191 to 1.839 kg and as expected, in all cases the allele derived from sika conferred lower carcass mass. The sika population was fixed for all small carcass mass alleles, whereas the red deer population was typically polymorphic. GO term analysis of genes lying in the QTL regions are associated with oxygen transport. Although body mass is a likely target of selection, none of the SNPs marking QTL are introgressing faster or slower than expected in either direction.

## Introduction

A major goal of evolutionary genetics is to understand the relationship between phenotypic and genetic variation. By understanding the genetic architecture of phenotypic traits, we can then ask how selection could act on a trait, make predictions of how a trait might change over time, or how the trait could respond to environmental change (Barton and Keightley 2002). In the context of hybridization, it is informative to understand the genetic architecture of the phenotypic traits that differ between hybridizing species. This is particularly relevant when human influences lead to increased hybridization (Grabenstein and Taylor 2018) and there is the potential for extinction via hybridization to decrease biodiversity (Rhymer and Simberloff 1996; Brennan *et al.* 2015; Todesco *et al.* 2016).

Genetic mapping in hybrid zones is particularly powerful because of the opportunity to use admixture mapping on recombinant individuals (Rieseberg and Buerkle 2002). The assumption of admixture mapping is that hybrid individuals have mosaic genomes that have been formed as the result of introgression, selection, recombination, and genetic drift (Buerkle and Lexer 2008; Winkler *et al.* 2010; Seldin *et al.* 2011). Coupled with divergent phenotypes, this allows for quantitative trait locus (QTL) mapping using fewer markers and individuals than are needed for typical genome-wide association studies (Rieseberg and Buerkle 2002). Natural hybrid zones can be extremely powerful for detecting QTLs when both the phenotype and genotypes are divergent between the two parental populations and when there are individuals sampled across the ancestry and phenotype spectrum (Buerkle and Lexer 2008).

Admixture mapping has been used in human populations, wild plants, and in some wild animals, but less so in wild mammals. Specifically, admixture mapping has been used extensively to find genes for: disorders in human populations (Patterson *et al.* 2004; Smith *et al.* 2004; Shriner 2013), reproductive isolation, morphological, and phytochemical traits in *Populus* hybrid zones (Lexer *et al.* 2007; Lexer *et al.* 2010; Bresadola *et al.* 2019), plumage color, migration behavior, and beak size in birds (Chaves *et al.* 2016; Delmore *et al.* 2016; Brelsford *et al.* 2017), melanoma and tail fin morphology in swordtail fish (*Xiphophorus malinche* and *Xiphophorus* *birchmanni*; Powell *et al.* 2020, 2021), and wing pattern variation in butterflies (Lucas *et al.* 2018). In wild mammal systems, admixture mapping has been used to discover 10 genomic regions for craniofacial shape variation and 23 single nucleotide polymorphisms (SNPs) associated with leg bone length in mice (*Mus musculus musculus x M. m. domesticus*; Pallares *et al.* 2014; Škrabar *et al.* 2018), and to associate introgressed genomic regions with body size and skeletal growth in coyotes and wolves (*Canus latrans and Canus* *lupus*; vonHoldt *et al.* 2016). Admixture mapping is suitable for QTL mapping in wild mammals, and systems with substantial divergence in focal phenotypes have the most power to detect associated markers.

Anthropogenic hybridization between red deer (*Cervus elaphus*) and sika (*Cervus* *nippon*) in Scotland (Senn and Pemberton 2009; McFarlane *et al.* 2020) offers an opportunity to use admixture mapping to identify the genetic architecture of an extremely variable phenotype, in this case, carcass mass. Briefly, sika were introduced to Scotland in the 19th century, and hybrid individuals are common in Kintyre (McFarlane *et al.* 2020). Carcass mass of red deer males in Argyll ranges between 55 and 106 kg, while carcass mass of red deer females ranges from 51 to 61 kg; by comparison sika in Scotland have an average carcass mass of 30 kg (males) and 24 kg (females; Harris and Yalden 2008) indicating substantial divergence in this trait between the two species. Hybrid individuals have intermediate phenotypes correlated with their admixture proportion (Senn *et al.* 2010). Carcass mass is the weight in kilograms of the animal at death, following the removal of the head, internal organs, lower legs, and blood. Thus, carcass mass is approximately 60–70% of live mass (Mitchell and Crisp 1981). Previous work in this system has used a 45K Illumina SNP chip to identify hybrid individuals with high confidence, pinpoint 629 diagnostic markers (fixed differences between species) and 3205 ancestry informative markers (extreme differences between species; McFarlane *et al.* 2020). Additionally, Bayesian genomic clines, which can be used to compare the rate and extent of introgression in hybrid populations (Gompert and Buerkle 2011; Gompert and Buerkle 2012), were used to quantify locus-specific introgression. We found that red deer and sika in Scotland are quite genetically diverged, with a genome-wide *F*_st_ of 0.532 (95% confidence interval: 0.529–0.534; McFarlane *et al.* 2020), although it should be noted that there is substantial variation in divergence across the genome (McFarlane *et al.* 2021). We also found substantial variation in the rate of introgression, indicating the potential for some SNPs to be under selection (McFarlane *et al.* 2021). If there is an SNP for carcass mass that is in a causal region, or in linkage disequilibrium (LD) with a causal region, we should have high power to detect it, based on the *F*_st_ between red deer and sika, the large phenotypic divergence and the estimated number of generations since admixture began (approximately 6–7; Crawford and Nielsen 2013; McFarlane *et al.* 2020).

The goals of this study are to use the red-sika hybrid system to (1) identify large-effect QTLs for carcass mass, (2) estimate the direction of effect of any QTL found, with the prediction that alleles associated with low mass would be at high frequency in sika and lower frequency in red deer, and (3) search for nearby genes and analyze their putative functions. In general, we expect carcass mass in deer to have a polygenic architecture, as is the case in morphological traits in several wild systems (*e.g.*, Soay sheep; Bérénos *et al.* 2015), collared flycatchers and house sparrows (Silva *et al.* 2017), and great tits (Santure *et al.* 2013, 2015). However, there could also be some large-effect QTLs, as have been found for human height (Yang *et al.* 2010), cattle (Bhuiyan *et al.* 2018; Hay and Roberts 2018; Pegolo *et al.* 2020), and such QTL are particularly likely to be detected in an admixed population.

## Methods

**Figure 1:**
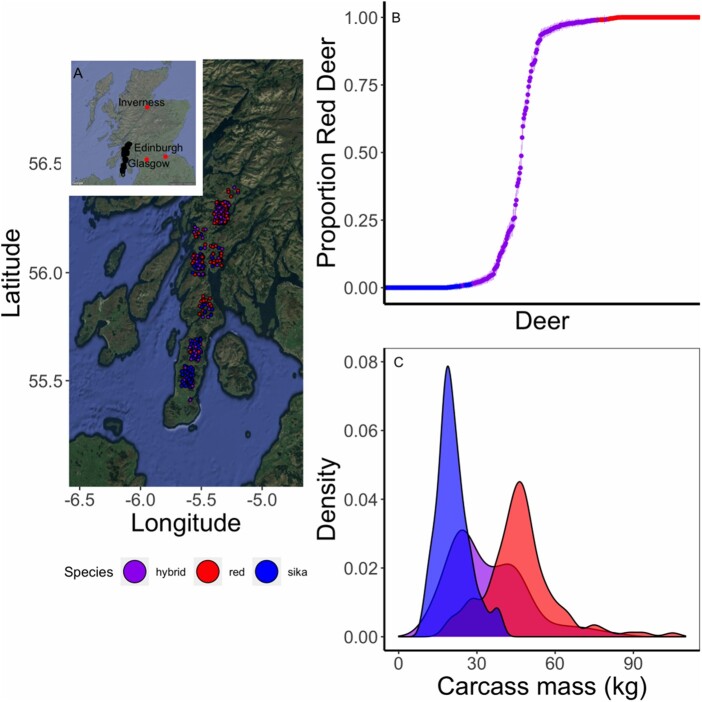
(A) Sampling sites of red deer (in red), sika (in blue), and hybrids (in purple) on the Kintyre Peninsula in Scotland. While 386 were sampled and weighed, the points for many deer overlap on this map. The inset is a map of Scotland, where sampling points are in black, and Edinburgh is marked in red. These maps are from Google Maps, accessed using ggmap (Kahle and Wickham 2013). (B) Estimates of admixture proportion (Q score) and 95% confidence intervals for individual deer from Kintyre with carcass weight measurements. This plot is modified from Figure 2 in McFarlane *et al.* (2020), but includes only those individuals with phenotypic measurements. Hybrid deer (*i.e.*, those with CIs that overlap neither 0 nor 1) are in purple, red deer (CIs overlap 1) are in red and sika (CIs overlap 0) are in blue. (C) Kernal smoothed density plot of red deer (in red), sika (in blue), and hybrids (in purple) carcass mass in kilogram. Carcass mass is between 60% and 70% of live mass.

We analyzed 513 deer samples collected from 15 forestry sites in the Kintyre region of Scotland between 2006 and 2011. The Forestry Commission Scotland (now Forestry and Land Scotland) culled the deer as part of normal deer control measures, in which animals were shot as encountered, regardless of phenotype or suspected species (Smith *et al.* 2018). Ear tissue samples were stored in 95% ethanol, and animals were sexed, aged (from tooth eruption and wear), and weighed to the nearest kg within 24 h of harvest (Senn and Pemberton 2009). Of the 513 deer sampled and genotyped, carcass mass was available for 386 animals. Only these 386 animals with phenotypes were used in all downstream analyses. The sampling site of each individual, identified by species (see below) is mapped in Figure 1A.

### DNA extraction and SNP genotyping

The deer were genotyped on the Cervine Illumina iSelect HD Custom BeadChip, which has 53,000 attempted SNP assays, using an iScan instrument (Huisman *et al.* 2016). When this SNP chip was developed, SNPs were selected to be spaced evenly throughout the genome based on the bovine genome with which the deer genome has high homology, although we use the deer linkage map in the present study (Johnston *et al.* 2017). The majority of SNPs were selected because they were polymorphic in red deer, specifically those red deer that are part of a long-term monitoring project on the Isle of Rum, but 4500 SNPs were also selected to be diagnostic between either red deer and sika or red deer and wapiti (*Cervus canadensis*; Brauning *et al.* 2015).

We used the DNeasy Blood and Tissue Kit (Qiagen) according to the manufacturer’s instructions to extract DNA for SNP analysis, with the exception that we eluted twice in 50-μl buffer TE to obtain DNA at a sufficiently high concentration. We assayed the concentration of extractions using the Qubit™ dsDNA BR Assay Kit (Invitrogen). If an extraction was below 50 ng/μl, it was vacuum-concentrated, re-extracted, or omitted from SNP analysis. Each 96 well plate had a positive control, and genotypes were scored using the clusters from a previous study (Huisman *et al.* 2016; McFarlane *et al.* 2020).

We followed the same protocol as McFarlane *et al.* (2020) for quality control, and to estimate the proportion of red deer ancestry for each individual (Q score). We used PLINK for all quality control (Purcell *et al.* 2007). Specifically, we excluded individual samples with a call rate of less than 0.90, deleted loci with a minor allele frequency of less than 0.001, and/or a call rate of less than 0.90 (McFarlane *et al.* 2020), but we did not exclude SNPs based on Hardy–Weinberg Equilibrium (HWE) as admixed samples are not expected to be in HWE. Species was assigned using ADMIXTURE in (Alexander *et al.* 2009; McFarlane *et al.* 2020). If the credible interval (CI) around the Q score overlapped 0, an individual was considered pure sika, if the CI overlapped 1 then it was pure red deer and the individual was considered a hybrid if the CIs overlapped neither 0 or 1 (McFarlane *et al.* 2020). Of the 386 individuals with phenotypes, 124 were red deer, 105 were sika, and 157 were hybrids (Figure 1B).

### Admixture mapping

We used Bayesian sparse linear mixed models (BSLMMs) in *gemma* for admixture mapping (Zhou *et al.* 2013). BSLMMs model the genetic architecture of traits while controlling for relatedness, thus giving an estimate of the proportion of phenotypic variance explained by combined effects of a polygenic distribution of all SNPs including large effects for some SNPs. SNP effects are drawn from two distributions, one distribution where it is assumed that all SNPs have a small to negligible effect, and a second distribution where some SNPs are assumed to have a larger effect drawn from a different distribution (*i.e.*, the sparse effects; Zhou *et al.* 2013). BSLMMs include a kinship matrix as a random effect to account for phenotypic similarity based on overall relatedness or genetic similarity. Inclusion of this kinship matrix removes the effect of population structure when determining whether individual SNPs have a significant effect on the trait (Zhou *et al.* 2013). From the BSLMM models, we can extract estimates of the proportion of variance in the phenotype explained (PVE) by the sparse effects and the kinship matrix and polygenic effects (*i.e.*, random effects), as well as the proportion of the genetic variance explained (PGE) by the sparse effects. The product of PVE and PGE is the proportion of phenotypic variance explained by the sparse effects, which can be interpreted as the narrow-sense heritability (*h*^2^; Bresadola *et al.* 2019), or the proportion of phenotypic variance explained by those SNPs with large-effect size (Zhou *et al.* 2013).

A BSLMM cannot be run with a covariate matrix, although covariates can be included as additional fixed effects in the above-mentioned SNP matrices using the command “–not-snp.” We added covariates to the input file, specifically a “bimbam dosage” file output using plink (Purcell *et al.* 2007; Bresadola *et al.* 2019). Because body mass in deer is known to be strongly influenced by age and sex (Clutton-Brock *et al.* 1982), we ran the BSLMM including these as additional covariates (age fitted as age in years). We also included the point estimate of Q score from ADMIXTURE (see above) as an additional covariate to account for background species differences (Pallares *et al.* 2014). We report the results of BSLMMs run both with and without the covariates. The BSLMM was run for 25 million iterations, with a burn-in of 10 million iterations, and sampled every 1000 iterations after the burn-in. Convergence was confirmed using plots of the MCMC distributions of PVE, PGE, and gamma (*i.e.*, the number of SNPs included in the sparse distribution), following Soria-Carrasco (2019). The model was run three times to ensure that a global peak was found. To determine significance, we quantified the posterior inclusion probability (PIP), and with a threshold of 0.1; those SNPs with a PIP higher than 0.1 are considered significantly associated with the phenotype (Chaves *et al.* 2016). We report in the main text all SNPs that we found to be significant in any of the all three runs of the model, and report the different effect sizes and PIPs from each run in Supplementary Table S1. We report exact estimates of PVE, PGE, and PIPs from the first run of the model (A in Supplementary Table S1), as all estimates were highly consistent. Finally, we do not report any effects for SNPs on the X chromosome, as we did not specifically account for different copy numbers between males and females, and did not have a sufficient sample size to run males and females in separate models.

To understand how genotypes for each highlighted SNP were associated with carcass mass, we used ADMIXTURE to determine the posterior population allele frequency in the parental red deer and sika populations, and to assign a “sika” and a “red deer” allele(s) (Alexander *et al.* 2009). We then plotted SNP genotypes for each sex against carcass mass after accounting for age.

### Gene enrichment analysis

To identify possible genes associated with carcass mass in red deer and sika, we first quantified the average LD across each linkage group in each of red deer, sika, and hybrids (as defined in McFarlane *et al.* 2020), using PLINK (Purcell *et al.* 2007). We used biomaRt (Durinck *et al.* 2005, 2009) and ensembl (Yates *et al.* 2020) to identify genes 500 kb up or downstream of the SNPs of interest, based on the high LD, we expect at this range. We also used biomaRt and ensembl to infer putative function of these genes in other organisms, specifically cattle and humans. Finally, we used g:Profiler for functional gene enrichment analysis, searching for relationships between gene in predefined gene sets, where genes are categorized together based on biochemical pathways, or consistent coexpression (Subramanian *et al.* 2005; Raudvere *et al.* 2019). We compared the identified genes and associated GO terms to the databases for cattle and humans. To account for multiple testing, we used a Benjamini–Hochberg FDR and examined each biological process (BP), molecular function (MF), and cellular component (CC) GO terms.

## Results

The red deer that we sampled had an average carcass mass of 37.2 kg (±17.9 SD, females, 39.5 kg ±24.2, males) while the sika weighed 19.4 kg (±4.3, females, 19.3 kg ±9.3 males), and hybrid individuals were intermediate at 22.7 kg (±16.8, females, 27.1 kg ± 21.5 males). The substantial variation within each sex species is due to variation in age (Figure 1B).

We estimated PVE, which includes sex, age, admixture proportion (Q), and the SNP effects (including the alpha matrix that included all SNP effects and the sparse matrix with the additional, large SNP effects) to explain 0.912 [0.87–0.95 (CI)] of the phenotypic variance in carcass mass, while PGE, the genetic variance due to the sparse effects was 0.656 (0.22–0.99). This means that 0.598 (0.19–0.94) of the phenotypic variance was explained by the sparse effects [*i.e.*, those effects due to SNPs with large effects, PGE/(PVE+PGE+residual); Bresadola *et al.* 2019). The sparse effects included sex and age, which both had extremely high PIP (PIPs of 1.00, 0.99, respectively) but not Q, which had a low PIP (0.0015).

**Figure 2:**
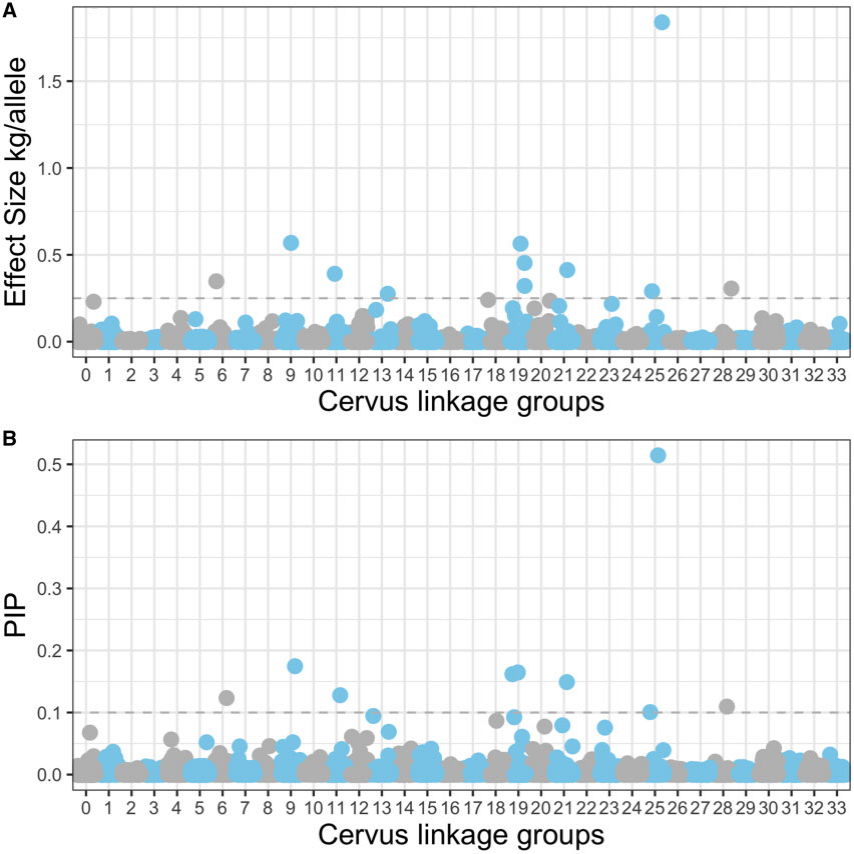
Effect size (A) and PIP (B) for each SNP in the sparse distribution across 33 *Cervus* linkage groups in an analysis of carcass mass, where group 0 are SNPs that are unmapped. We have not included the X chromosome. Age, sex, and Q score were included in this analysis. SNPs that were included in the sparse distribution at least 10% of the time (those above the grey-dashed line) are considered significant (see Table 1).

The mean number of SNPs included in the sparse effects was 58.1 (6–179), but only 9 (10 in run C, Supplementary Table S1) SNPs had a PIP above the threshold of 0.1 (*i.e.*, these SNPs were included in the sparse effect distribution at least 10% of the time). These SNPs were on linkage groups 6, 9, 11, 19, 21, 25, and 28. All SNPs with a PIP higher than 0.1 were also in the 99.9th percentile of effect sizes (Table 1; Figures 2, A and B), and some are clustered, for example, those on linkage group 19.

**Table 1:** SNPs that were included in the sparse distribution at least 10% of the time (*i.e.*, PIP equal or more than 0.10)

Red deer linkage group	SNP name	Effect Size	PIP	Sika Allele	Sika allele Frequency (in sika)	Red deer major allele	Major allele frequency (in red deer)	Red deer minor allele	Minor allele frequency (in red deer)	V_SNP_ and *h*^2^_SNP_
6	cela1_red_6_92593028	0.348	0.124	G	1.000	A	0.541	G	0.459	0.048 (0.00019)
9	cela1_red_7_76865763	0.569	0.175	A	1.000	G	0.771	A	0.229	0.086 (0.00033)
11	cela1_red_11_67732563	0.391	0.128	C	0.995	C	0.621	A	0.379	0.068 (0.00027)
19	cela1_red_1_65547414	0.453	0.165	A	0.990	G	0.627	A	0.373	0.072 (0.00028)
19	cela1_red_1_62769154	0.564	0.162	A	0.990	A	0.850	G	0.150	0.159 (0.00062)
20	cela1_red_3_12407635*	0.191	0.078	G	0.995	G	0.519	A	0.481	0.016 (0.00006)
21	cela1_red_14_44586160	0.412	0.149	A	0.990	A	0.506	G	0.494	0.067 (0.00062)
25	cela1_red_20_30400180	1.839	0.515	C	1.000	A	0.691	C	0.309	1.030 (0.00400)
25	cela1_red_20_41494052	0.290	0.101	G	1.000	A	0.771	G	0.229	0.024 (0.00009)
28	cela1_red_9_19132996	0.306	0.110	A	1.000	A	0.516	G	0.484	0.038 (0.00015)

One SNP (cela1_red_3_12407635, noted with an *) only had a PIP above 0.1 in one of the three replicate runs of GEMMA (Supplementary Table S1). We report here the effect size (in kg) and the PIP. We also note the major allele in sika and in red deer, and the allele frequency of these alleles in the parental species, as well as the minor allele and frequency in red deer. While the sika alleles are nearly fixed in sika for these SNPs, red deer are polymorphic for nearly all SNPs. Finally, we estimated the variance in carcass mass explained by each SNP, using the additive-only effect formula V_SNP_ = 2pqa^2,^ where p is the minor allele frequency for the whole red deer, sika, and hybrid system, q is 1-p and a is the effect size. The SNP specific heritability (*h*^2^_SNP_), in parentheses, is the V_SNP_ divided by the phenotypic variance (V_P_).

**Figure 3:**
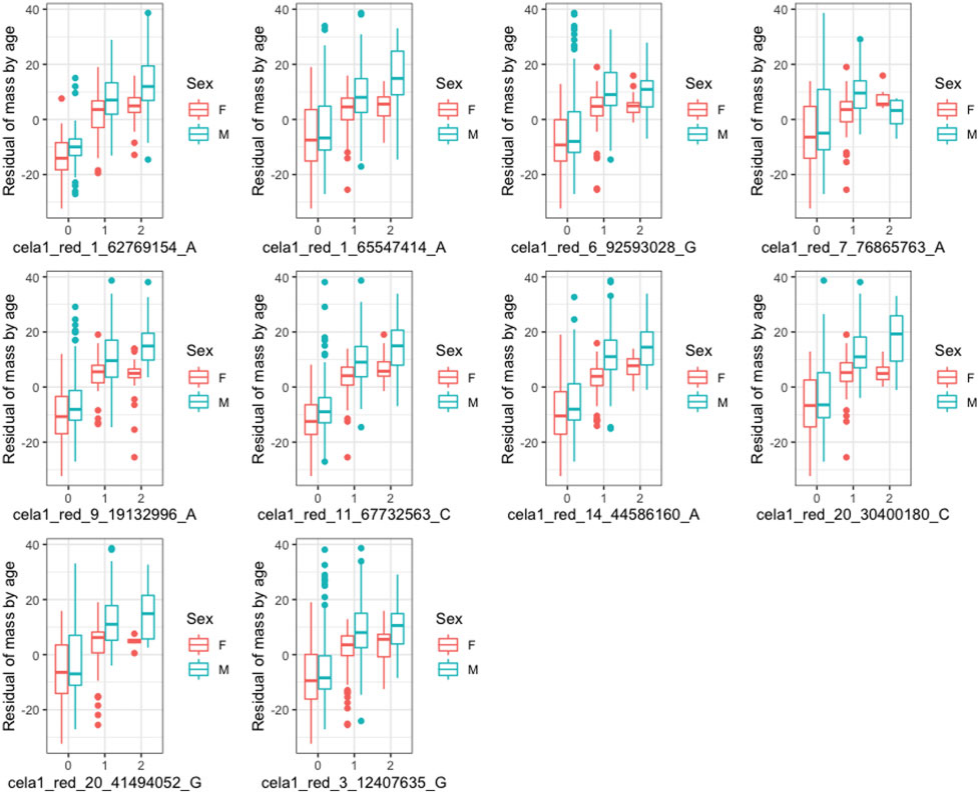
Box and whisker (central line = median, boxes 25–75%, lines 5–95%) plots illustrating the relationship between the genotypes of each SNP that was included in the sparse distribution at least 10% of the time and carcass mass of each male and female deer. The *x*-axis label notes the SNP name. “0” indicates homozygotes for the sika allele, 1 heterozygote for the sika allele and a red allele, and 2 homozygotes for the red allele. Carcass weights have been regressed against age, and then plotted in each sex separately. A similar plot of the raw mass (instead of the residual mass) can be found in the Supplementary material (Supplementary Figure S2).

To examine whether those alleles associated with small size were more prevalent in sika, we categorized alleles as sika or red deer alleles, based on posterior estimates of parental population allele frequencies from ADMIXTURE. Essentially, we characterized fixed or nearly fixed alleles in the pure sika population as the “sika” allele, and identified the “red deer allele” depending on allele frequency in the pure red deer population (*i.e.*, the major allele in red deer could be the same as the sika allele). For all SNPs associated with carcass mass, we found that, as predicted, the allele for lower carcass mass was fixed or nearly fixed in sika, and polymorphic in red deer (Figure 3 and Table 1).

We found an average within-linkage group LD of 0.425 ± 0.26 SD in red deer, 0.435 ± 0.22 in hybrid deer and 0.781 ± 0.26 in sika, which varied across linkage groups (Supplementary Figure S1). From this high LD, we conservatively inferred that genes within 500 kbp of each of the significant SNPs could be related to carcass mass. We found 45 unique genes that have been named in the cattle genome (Supplementary Table S2), and 297 unique GO terms (Supplementary Table S3). We found 15 GO terms that were significantly associated via gene set analysis based on the genes that we identified (Table 2; Supplementary Table S4). We found qualitatively similar interactions when we assessed the GO terms and interactions in humans, although without any significant GO:BP interactions, and without any three-gene interactions (Supplementary Table S4).

**Table 2:** Identified, significant gene ontology terms that are enriched across genes near the SNPs associated with carcass mass in red deer and sika when compared to the cattle genome

Source	GO term name	GO term_id	Adjusted *P*-value	-Log10 *P*-value	Intersection size	Gene interactions
GO:MF	Oxygen carrier activity	GO:0005344	0.000	3.520	3	HBM, HBA, HBQ1
GO:MF	Oxygen binding	GO:0019825	0.000	3.514	3	HBM, HBA, HBQ1
GO:MF	Molecular carrier activity	GO:0140104	0.004	2.368	3	HBM, HBA, HBQ1
GO:MF	Alkylbase DNA N-glycosylase activity	GO:0003905	0.022	1.652	1	MPG
GO:MF	DNA-3-methyladenine glycosylase activity	GO:0008725	0.022	1.652	1	MPG
GO:MF	Heme binding	GO:0020037	0.022	1.652	3	HBM, HBA, HBQ1
GO:MF	DNA-3-methylbase glycosylase activity	GO:0043733	0.022	1.652	1	MPG
GO:MF	DNA-7-methylguanine glycosylase activity	GO:0043916	0.022	1.652	1	MPG
GO:MF	Tetrapyrrole binding	GO:0046906	0.022	1.652	3	HBM, HBA, HBQ1
GO:MF	DNA-7-methyladenine glycosylase activity	GO:0052821	0.022	1.652	1	MPG
GO:MF	DNA-3-methylguanine glycosylase activity	GO:0052822	0.022	1.652	1	MPG
GO:MF	2,4-dienoyl-CoA reductase (NADPH) activity	GO:0008670	0.041	1.389	1	DECR2
GO:BP	Gas transport	GO:0015669	0.001	3.008	3	HBM, HBA, HBQ1
GO:BP	Oxygen transport	GO:0015671	0.001	3.008	3	HBM, HBA, HBQ1
GO:CC	Hemoglobin complex	GO:0005833	0.000	3.753	3	HBM, HBA, HBQ1

Possible gene ontology sources are GO: Molecular Function (GO:MF), GO: Biological Processes (GO:BP), and GO: Cellular Components (GO:CC). The association between the identified genes noted in Gene Interactions and the identified SNPs in this study can be found in Supplementary Table S2.

## Discussion

Red deer and sika differ substantially in carcass mass, while hybrid deer are intermediate in mass (Senn *et al.* 2010). We have identified 10 autosomal SNPs that are related to carcass mass, which are associated with 7 chromosomes, 45 genes, and 297 GO terms. Our use of an anthropogenic hybrid swarm for admixture mapping has illuminated potential candidate regions in red deer and sika, which could explain variation in mass in other deer species, or even other mammalian systems.

We found that a large proportion of the phenotypic variation was explained by the sparse effect 0.598 (CI 0.19–0.94). A substantial proportion of the phenotypic variance must be due to including sex, age in the analysis, as sex and age were consistently included in the sparse effect. However, a moderate heritability is intuitive, as body mass is a moderately heritable trait [average *h*^2^ for wild animal body mass/weight 0.371 ± 0.26 (SD); Postma 2014]. While we found sex and age were consistently included in the sparse effects, Q score was not. This is surprising, as Q has been shown to predict carcass mass in this population previously (Senn *et al.* 2010). However, as GEMMA includes a kinship effect in the BSLMM, and kinship and Q are strongly correlated (Supplementary Figure S3), it is likely that the variance that would otherwise be explained by Q was captured by the kinship effect. We found that the significant SNPs each explained between 9.4e^−5^ and 4.0e^−3^% of the variation in carcass mass (Table 1). Finally, it should be noted that 386 individuals are a relatively small sample size, which could lead to the overestimation of PVE and PGE.

We have identified a number of candidate regions that are associated with carcass mass in red deer, sika, and their hybrids. The 10 autosomal SNPs that we have identified are all extremely invariant in sika (sika allele frequency > 0.99), but polymorphic in red deer, as determined by ADMIXTURE (Alexander *et al.* 2009; McFarlane *et al.* 2020). In every case, the allele that was fixed in sika was associated with smaller size (Figure 3). Additionally, based on substantial LD across each linkage group (Table 2), we identified 45 genes that could be functionally associated with carcass mass in deer (Supplementary Table S2). For context, none of these genes have been previously associated with carcass mass in white-tailed deer (Anderson *et al.* 2020) or cattle (cow QTL database; Bouwman *et al.* 2018; Hu *et al.* 2019), 4 of them are associated with height in humans (Locke *et al.* 2015) and 12 are associated with body mass index or obesity in humans (Comuzzie *et al.* 2012; Danjou *et al.* 2015; Winkler *et al.* 2015; Wojcik *et al.* 2019). Perhaps the strongest evidence we have for carcass mass QTL is where multiple adjacent SNPs indicate an effect. We found multiple SNPs on linkage groups 19 and 25, and in both cases, these SNPs have large-effect sizes as well as significant PIPs (Figure 2, A and B). We found 25 genes within 500,000 bp of the two SNPs identified on linkage group 25, and 6 of these genes were part of predetermined gene sets, with associated GO terms (Table 2). Specifically, *HBM*, *HBA*, and *HBQ1* are all found on linkage group 25 and are associated with oxygen binding and transport (Table 2). Future functional work could explore if oxygen binding and transport influence growth in deer.

There are a number of genomic investigations that could follow up this study. For example, we could use phasing to examine whether haplotype frequencies at each of these loci differ between red deer and sika. It would also be interesting to look at sequence divergence between red deer and sika for each of the 45 genes that we have identified here. While there is a sika genome (Xing *et al.* 2021) and a Hungarian red deer genome (Ababaikeri *et al.* 2020), neither of these is from Scottish red deer nor Scottish sika, which would be ideal for comparing divergence within this system, particularly given that introgression is occurring and selection is possible. Additionally, future work could look at gene expression at these genes to estimate whether expression differences are correlated with carcass mass (Todd *et al.* 2016). These additional methods would be useful to validate the present findings.

It would be interesting to quantify selection on the specific SNPs that we have found here, to determine the potential for these genomic regions to respond to selection on body size. In a previous study, we used genomic clines in the program *bgc* to look for SNPs that could be associated with either reproductive isolation or adaptive introgression (*i.e.*, those SNPs are introgressing either slower or faster than the genome average; Gompert and Buerkle 2011; Gompert and Buerkle 2012; McFarlane *et al.* 2021). We have previously reported that 11.4% of SNPs have cline centers more extreme than the genome average and 17.6% of SNPs have introgressed at rates more extreme than the genome average (McFarlane *et al.* 2021). However, we found that these patterns could be driven by either genetic drift or selection (McFarlane *et al.* 2021). For this reason, we wanted to determine if any QTLs associated with carcass mass had significant genomic cline parameters, as these would be independent analyses suggesting selection on specific SNPs. However, none of the SNPs associated with carcass mass are introgressing faster than the genome-wide expectation, although this does not eliminate the possibility of selection for carcass mass alleles within each population. Ideally, we would measure selection on the phenotypes of hybrid individuals with a variety of genotypes to make firm statements about selection on the carcass mass loci we have identified here, and then to make predictions about the potential for adaptive introgression (Taylor and Larson 2019). However, in lieu of directly measuring fitness, admixture mapping is one way to identify regions of the genome that are potentially contributing to introgression in hybrid systems, particularly for traits such as carcass mass which can be assumed to be under selection. It is because admixture mapping is so inherently powerful that we were able to identify SNPs explaining a substantial proportion of phenotypic and genetic variance in a quantitative trait in this wild deer system.

## Data availability

All data and scripts for this project can be found at https://figshare.com/projects/Admixture_mapping_reveals_loci_for_carcass_mass_in_red_deer_x_sika_hybrids_in_Kintyre_Scotland/112743. Supplementary material is available at figshare: https://doi.org/10.6084/m9.figshare.15057357.
